# Dengue Serosurvey in Sint Eustatius

**DOI:** 10.1371/journal.pone.0095002

**Published:** 2014-06-10

**Authors:** Teresa Leslie, Nicholas J. Martin, Carol Jack-Roosberg, George Odongo, Edwin Beausoleil, Jennifer Tuck, Kanakatte Raviprakash, Tadeusz J. Kochel

**Affiliations:** 1 Department of Anthropology, University of Maryland, College Park, Maryland, United States of America; 2 Eastern Caribbean Public Health Foundation, Sint Eustatius, Netherlands Dutch Caribbean; 3 Viral and Rickettsial Diseases Department, Naval Medical Research Center, Silver Spring, Maryland, United States of America; 4 Sint Eustatius Public Health Department, Sint Eustatius, Netherlands Dutch Caribbean; 5 Queen Beatrix Medical Center, Sint Eustatius, Netherlands Dutch Caribbean; University of Rochester, United States of America

## Abstract

Four distinct serotypes of dengue viruses (DENV) are the cause of re-emerging dengue fever (DF) and dengue hemorrhagic fever (DHF). Dengue circulation in the Caribbean has gone from none or single serotype to multiple serotypes co-circulating with reports of continuing cycles of progressively more severe disease in the region. Few studies have investigated dengue on Sint Eustatius. Blood samples were collected to determine the prevalence of antibodies against dengue in the Sint Eustatius population. Greater than 90% of the serum samples (184 of 204) were positive for anti-flavivirus antibodies by enzyme linked immunosorbance assay (ELISA). Plaque reduction neutralization test (PRNT), specific for dengue viruses, showed that 171 of these 184 flavivirus antibody positive sera had a neutralization titer against one or more DENV serotypes. A majority of the sera (62%) had neutralizing antibody to all four dengue serotypes. Only 26 PRNT positive sera (15%) had monotypic dengue virus neutralizing antibody, most of which (20 of 26) were against DENV2. Evidence of infection with all four serotypes was observed across all age groups except in the youngest age group (10–19 years) which contained only DENV2 positive individuals. In a multiple logistic regression model, only the length of residence on the island was a predictor of a positive dengue PRNT_50_ result. To our knowledge this is the first dengue serosurveillance study conducted on Sint Eustatius since the 1970s. The lack of antibodies to the DEN1, 3, and 4 in the samples collected from participants under 20 years of age suggests that only DEN2 has circulated on island since the early 1990s. The high prevalence of antibodies against dengue (83.8%) and the observation that the length of time on the island was the strongest predictor of infection suggests dengue is endemic on Sint Eustatius and a public health concern that warrants further investigation.

## Introduction

Dengue is a vector-borne disease that reemerged in the Americas during the latter half of the 20^th^ century [1], [2]. Transmitted by female mosquitoes of the genus *Aedes*, dengue is caused by infection with one of the four dengue viruses (DEN-1 through DEN-4) belonging to the family *Flaviviridae* genus *flavivirus*. Dengue infection generates a broad spectrum of clinical illness ranging from asymptomatic infections, mild dengue fever (DF), to severe and life-threatening disease including dengue hemorrhagic fever (DHF) and dengue shock syndrome (DSS).

The World Health Organization (WHO) reported over two million confirmed cases of dengue fever in 2010, of which more than 4,000 were fatal [2]. The number of reported cases may not represent the true burden of disease; approximately 40% of the global population lives in close proximity of the disease vector in more than 125 countries in Africa, the Americas, the eastern Mediterranean, Asia, and the Western Pacific. Infection with DEN is now spreading to new areas causing outbreaks and is estimated to infect 50–100 million people resulting in 500,000 cases of DHF or DSS [2], [3].

The Caribbean is rapidly becoming a dengue hyperendemic region with continuing cycles of progressively more severe dengue and DHF epidemics [4]. In the Caribbean, serotype circulation has gone from none or single serotype to multiple serotypes co-circulating [1]. This change in dengue virus circulation patterns and the increase in DHF cases have led to warnings of increased risk to travelers and local populations by national and inter-national public health agencies. Historically, dengue-like outbreaks have been reported in the Americas, including the Caribbean, as early as mid-19^th^ century. However, in the Caribbean, the first isolation of dengue-2 virus from the blood of a patient in Trinidad was reported only in 1953 [5]. Epidemics in Jamaica, Puerto Rico and the Lesser Antilles in the 1960's have been reported [6]–[8]. Throughout the Caribbean region, 700,000 cases of DF and 6,000 cases of DHF were recorded in 1995 and 1997 [9]–[12]. The rate of dengue in Jamaica has almost doubled every year since 2005 and in both Trinidad and Barbados there has been an increase in the outbreaks of dengue, where the disease is now considered endemic [4]. Notwithstanding, a recent metaanalysis by Brady et al concluded that with only 22% of dengue-present Caribbean countries displaying dengue data publicly, dengue status in these small island nations appeared more heterogeneous [13].

To date, there have been few studies investigating dengue in the Dutch Caribbean islands (formerly Netherland's Antilles), specifically on the island of Sint Eustatius. Disease vector surveys conducted in the 1940's and 1980's reported the presence of *Aedes aegypti* on the island [14]–[16]. The last dengue sero-prevalence study of Sint Eustatius, published in 1979 observed antibodies to Group B viruses (DEN1, DEN2, DEN3, lileus, yellow fever and Saint Louis Encephalitis) in 91% of those surveyed [17]. A distinct age distribution was observed, the percent of samples with antibodies against Group B arboviruses gradually rising from 86% in the 0–9 year age group to 100% in persons over 70 years of age. No serum tested during this study was monotypic to one dengue serotype, suggesting exposure to multiple dengue serotypes had occurred on the island. Continuous island specific dengue disease data are not available; however epidemiologic data compiled for the entire region indicate that outbreaks of DEN2 and DEN3 have occurred on islands adjacent to Sint Eustatius [18], [19].

In this study, a cross sectional design was utilized to assess DEN seroprevalance in Sint Eustatius. Sero-prevalence was established by initial screening of samples using dengue IgG enzyme linked immunosorbance assay (ELISA), followed by further testing for virus neutralizing antibody using the Plaque Reduction Neutralization Test (PRNT). Additionally, participants were grouped by age to understand the temporal dynamics of dengue infections in Sint Eustatius.

## Materials and Methods

### Ethics Statement

The study protocol was approved by the University of Maryland Institutional Review Board (11-0059) and Naval Medical Research Center Institutional Review Board (NMRC.2011.0011). All participants provided written informed consent to participate in the study. Sample collections under the study protocol were in compliance with all applicable Federal regulations governing the protection of human subjects.

### Study Area

Our study was conducted on Sint Eustatius, a hilly island with a dormant volcano located in the Eastern Caribbean (17°28′N, 62°58′W). There are 23 neighborhoods identified by residents and the government, which can be grouped into five regions based on their location on the island. The population of Sint Eustatius is approximately 3,500 individuals of diverse native and immigrant background. Sint Eustatius Department of Public Health records indicate the majority of the native population is of African and mixed African descent while the immigrant population is predominantly comprised of individuals from other Caribbean islands, China, and the Netherlands (unpublished data).

### Participants

Participants were recruited from the general population. Recruitment efforts were conducted at schools, churches and the two largest employers on the island (Government and NuStar Oil Terminal). Radio and television advertisements were used to inform people about the study and the importance of their participation. Individuals were asked to visit the Queen Beatrix Medical Center where they would be enrolled in the study. An individual trained to describe the study and administer the consent form would then enroll participants at the hospital.

### Sample Collection

All participants were asked to provide 3 mL of blood and complete a brief survey developed for this study. Blood collection was performed at the Queen Beatrix Hospital by trained medical professionals. The sera collection procedures were based on the Clinical and Laboratory Standards Institute (formerly NCCLS). Samples were allowed to clot at room temperatures (20–25°C) and then centrifuged according to the NCCLS guidelines, (Approved Standard- Procedures for the Collection of Diagnostic Blood Specimens by Venipuncture, H3-A4, 1998). Samples were stored at −20°C prior to shipment on ice to the Naval Medical Research Center, Silver Spring, MD for analysis. At the time of blood draw, self-reported history of dengue infection and demographic data were collected through a brief questionnaire that included multiple choice, binary and open ended responses.

### Sample Analysis

All sera were tested for the presence of anti-dengue IgG antibodies by ELISA as described previously [20] except that PEG-precipitated dengue virions (a mixture of all 4 serotypes) were used as the antigen and a peroxidase labeled anti-human Ig was used as the conjugate. A 1∶100 dilution of serum samples was used. If sera tested positive, they were further tested for neutralization of specific virus serotypes (DEN1: WESTEN PACIFIC 74, DEN2: OBS8041, DEN3: CH53489 and DEN4: 341750) using the PRNT as described previously [21] using two fold serial dilutions of serum samples. Vero cells were used for the assay and known negative and positive samples were used as controls. Highest dilution that resulted in a plaque reduction of 50% or more was reported as PRNT-50 titer. Based on previous experience, a titer of 120 was set as the cutoff threshold for a sample to be considered positive for a specific serotype.

### Statistical Analysis

Analysis of variance (ANOVA) was performed to determine if there was a significant difference between the prevalence of antibodies against specific dengue serotypes based on the location of residence, age, and length of time living on Sint Eustatius. A multiple logistic regression was performed to determine if age, years on the island, gender, or place of birth predicted the presence of antibodies against any dengue serotype. A Wald Chi-Square was performed for all predictors used in the multiple logistic regression to determine if any individual predictor's regression coefficient was not zero indicating statistical significance for that predictor.

## Results

### Participants

A total of 204 participants were enrolled in the study from October–December, 2011Due to the small size of the island and population, as well as the Queen Beatrix Medical Center being the only clinic on the island, sample collection from all participants was performed at this Medical Center. This convenient sampling strategy provided greatest access to the Sint Eustatius population. A summary of participant demography is given in Table 1.

**Table 1:** Participant demographics.

No. of Participants	204
**Average age** *	48.1±15.6 years
**Age range**	**(n)**
10–19	6
20–29	20
30–39	23
40–49	48
50–59	50
60–69	26
70–79	8
≥80	6
**Male** *	55
**Female**	104
**Average period lived on Sint Eustatius**	26.5±21.5 years
**Neighborhood** *	**(n)**
Golden Rock	59
Island Estates	19
Oranjstead	22
Union Estates	69
Zeelandia	3

*From those who answered questions related to age (n = 187), gender (n = 159) and location of residence (n = 172).

### Serology

**Figure 1:**
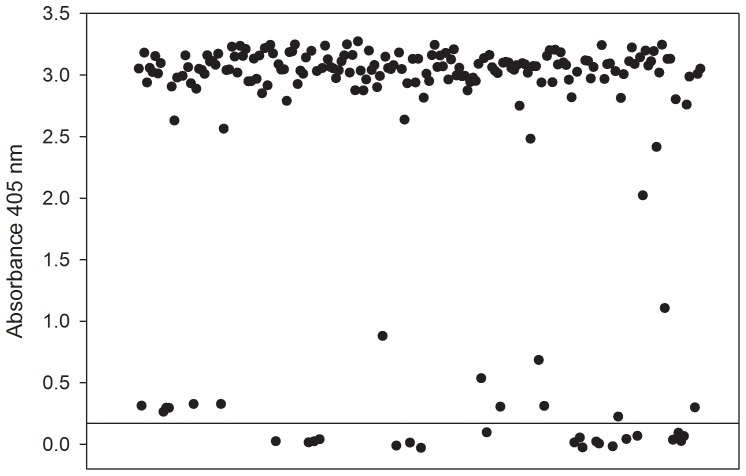
Anti-flavivirus antibody by ELISA. Serum samples (1∶100 dilution) were assayed by standard indirect ELISA using dengue (types 1–4) antigen coated microtiter plates. Antibody binding to negative antigen control was used to determine background and threshold for positive assays (heavy horizontal line). Each dot represents an individual.

Single-dilution (1∶100) ELISAs were performed on 204 samples collected from residents on Sint Eustatius. Of those, 184 samples (90.2%) tested positive for anti-flavivirus antibody (Figure 1). A large majority of the samples contained high levels of anti-flavivirus antibody (O.D. ≥2.5).

**Figure 2:**
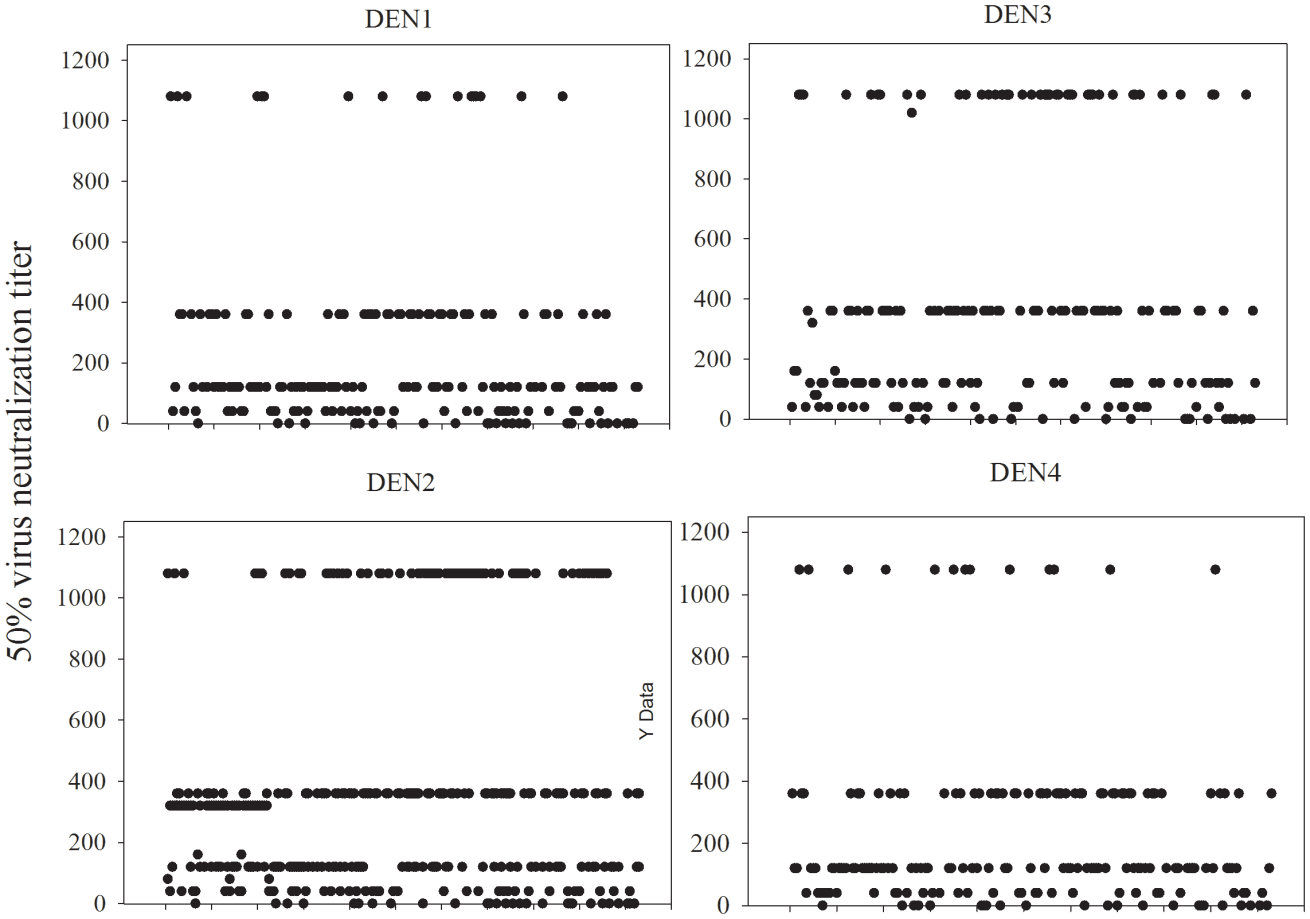
Dengue virus neutralizing antibody. Fifty percent virus neutralization titers against 4 serotypes of dengue virus (D1, D2, D3 and D4) were determined by standard plaque reduction neutralization test. A titer of 120 or greater for a particular serotype was considered positive for that serotype.

**Figure 3:**
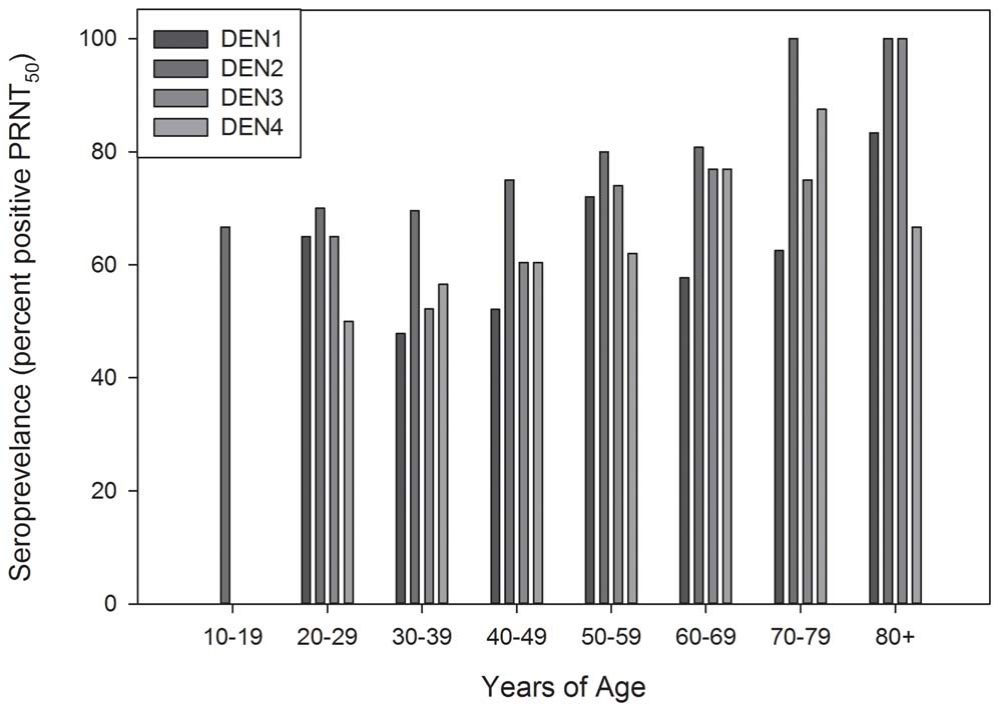
Dengue seropositivity as a function of age. All age groups except age group 10–19 (n = 6) showed evidence of exposure to all 4 dengue serotypes. All seropositive subjects in the 10–19 years group contained exclusively dengue-2 neutralizing antibody.

**Figure 4:**
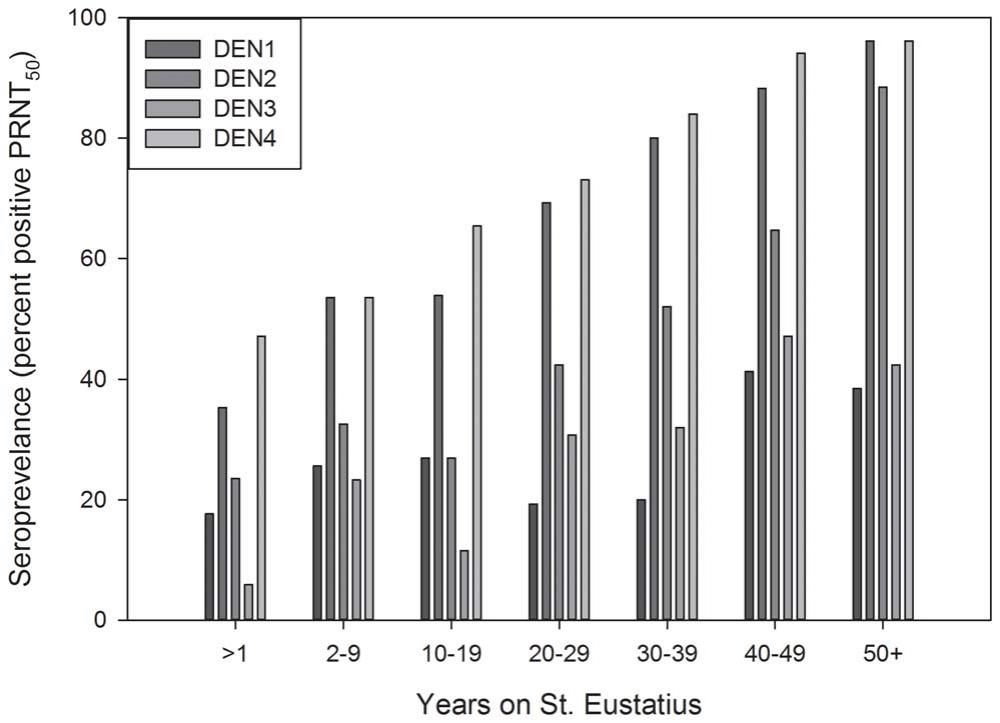
Dengue seropositivity as a function of self-reported time living on Sint Eustatius. ANOVA and Post Hoc analysis (Holmes-Sidack model) revealed significantly higher DEN seroprevalence in individuals living on the island for 50+ years group compared to all other groups except the 40–49 years. Additionally, significant higher seropositivity was noted between the individuals residing on the island for 40–49 years compared to those living on the island for 2–9 and 10–19 years.

Seropositive samples (n = 184) were tested for neutralization of dengue viruses (types 1–4) by a plaque reduction neutralization test (PRNT). Results (Figure 2) indicated that sera from a majority of individuals contained antibody that neutralized dengue viruses. A summary analysis (Table 2) showed that 106 (57.6%) individuals had antibodies that neutralized all 4 serotypes of dengue virus. Twenty six (14.1%) and twelve (6.5%) individuals had antibodies to 3 and 2 dengue serotypes, respectively and the remaining 27 individuals (14.7%) had monotypic antibodies. Interestingly, a majority of individuals with monotypic antibody (22 of 27) had only dengue-2 neutralizing antibodies (not shown). Analysis of participant age and dengue seropositivity showed evidence of infection with all four serotypes across all age groups over 20 years of age (Figure 3). In the age 10–19 group (n = 6) only antibodies against DENV2 were detected. The relative percent of DEN PRNT_50_ positive samples were stratified by region (see Table 1) on the island. There was no significant difference in DEN seroprevalence among individuals from different regions on the island (not shown, Kruskal-Wallis ANOVA on Ranks; H = 7.360 with 6 degrees of freedom; P = 0.289). This was expected due to the small size of Sint Eustatius island and no significant observable differences among the different regions in terms of socio-economic status, sanitation etc. However, a significant difference in DENV seroprevalence based on the length of time on Sint Eustatius was observed (Figure 4; ANOVA; F = 14.965; P<0.001). Post hoc analysis revealed significantly higher DEN seroprevalence in people who resided on the island for longer periods of time (Holm-Sidak method; P<0.05). Wald's Chi-Square test revealed that among factors such as age, gender, time lived on Sint Eustatius and location within the island, only number of years on Sint Eustatius had a significant contribution to the predicting the presence of antibodies against any dengue serotype (p<0.001). Although the group sizes are small and vary among groups, data suggest a possible association between dengue seropositivity and the length of time an individual has resided in Sint Eustatius.

**Table 2:** Seroprevalance of anti-dengue antibodies by virus neutralization (n = 184).

Exposure		Exposure	
DEN1	122 (66.3%)	One serotype	27 (14.7%)
DEN2	164 (89.1%)	Two serotype	12 (6.5%)
DEN3	139 (75.5%)	Three serotype	26 (14.1%)
DEN4	130 (70.7%	Four serotype	106 (57.6%)
DEN	171 (92.9%)		

## Discussion

This is the first dengue serosurveillance study conducted on Sint Eustatius in more than 30 years. A previous serosurvey, conducted in 1970s [17] concluded that DENV2 was endemic on the island with periodic epidemics of DENV1. Similar to this previous report, the results presented here suggest that the DENV2 is endemic and that DENV1, 3, and 4 are sporadically transmitted on the island (Table 1). Furthermore, the lack of antibodies to the DENV1, 3, and 4 serotypes in the samples collected from participants younger than 20 year of age suggests that these serotypes may not have circulated on the island since the early 1990s, despite isolation of DENV3 from patients during an outbreak on the adjacent island of St. Martin in 2003–2004 [18].

The lack of a significant difference in the seroprevelance of DEN between the five regions on the island was not surprising, given the small size and population (3,500) of Sint Eustatius. The length of time on the island was the only predictor of previous DENV infection, measured by the detection of serotype specific antibodies against DENV. This finding, coupled with the high prevalence (83.8%) of antibodies against one or more DENV serotypes suggests that dengue is endemic on Sint Eustatius.

This study surveyed a convenient population sample and may not reflect the dengue exposure history for all residents. Despite this limitation, dengue appears to be a real public health concern on Sint Eustatius that warrants further investigation. Future studies should focus on determining the dengue vector present on the island and continued characterization of the dengue transmission cycle unique to Sint Eustatius.
